# Plasma Nuclear Magnetic Resonance Metabolomics Discriminates Between High and Low Endoscopic Activity and Predicts Progression in a Prospective Cohort of Patients With Ulcerative Colitis

**DOI:** 10.1093/ecco-jcc/jjy101

**Published:** 2018-07-17

**Authors:** Fay Probert, Alissa Walsh, Marta Jagielowicz, Tianrong Yeo, Timothy D W Claridge, Alison Simmons, Simon Travis, Daniel C Anthony

**Affiliations:** 1 Department of Pharmacology, University of Oxford, Oxford, UK; 2 Translational Gastroenterology Unit, Oxford University Hospitals NHS Foundation Trust, Oxford, UK; 3 MRC Human Immunology Unit, Weatherall Institute of Molecular Medicine, University of Oxford, and Translational Gastroenterology Unit, John Radcliffe Hospital, Oxford, UK; 4 Department of Neurology, National Neuroscience Institute, Jalan Tan Tock Seng, Singapore; 5 Department of Chemistry, University of Oxford, Chemistry Research Laboratory, Oxford, UK

**Keywords:** ulcerative colitis, metabolomics, biomarker

## Abstract

**Background and Aims:**

Endoscopic assessment of ulcerative colitis [UC] is one of the most accurate measures of disease activity, but frequent endoscopic investigations are disliked by patients and expensive for the healthcare system. A minimally invasive test that provides a surrogate measure of endoscopic activity is required.

**Methods:**

Plasma nuclear magnetic resonance [NMR] spectra from 40 patients with UC followed prospectively over 6 months were analysed with multivariate statistics. NMR metabolite profiles were compared with endoscopic [Ulcerative Colitis Endoscopic Index of Severity: UCEIS], histological [Nancy Index] and clinical [Simple Clinical Colitis Activity Index: SCCAI] severity indices, along with routine blood measurements.

**Results:**

A blinded principal component analysis spontaneously separated metabolite profiles of patients with low [≤3] and high [>3] UCEIS. Orthogonal partial least squares discrimination analysis identified low and high UCEIS metabolite profiles with an accuracy of 77 ± 5%. Plasma metabolites driving discrimination included decreases in lipoproteins and increases in isoleucine, valine, glucose and *myo*-inositol in high compared to low UCEIS. This same metabolite profile distinguished between low [Nancy 0–1] and high histological activity [Nancy 3–4] with a modest although significant accuracy [65 ± 6%] but was independent of SCCAI and all blood parameters measured. A different metabolite profile, dominated by changes in lysine, histidine, phenylalanine and tyrosine, distinguished between improvement in UCEIS [decrease ≥1] and worsening [increase ≥1] over 6 months with an accuracy of 74 ± 4%.

**Conclusion:**

Plasma NMR metabolite analysis has the potential to provide a low-cost, minimally invasive technique that may be a surrogate for endoscopic assessment, with predictive capacity.

## 1. Introduction

In practice, a combination of symptoms, quality of life, blood tests, endoscopic and histopathological assessments are used to modify ulcerative colitis [UC] therapy on a case-by-case basis with the aim of achieving and maintaining remission. Clinical indices such as the Simple Clinical Colitis Activity Index [SCCAI]^1^ are used to monitor UC and offer the advantage that patients can complete the index independently, allowing more frequent and convenient monitoring. However, symptoms do not reliably correlate with the degree of underlying inflammation and one of the ultimate targets of treatment is to achieve endoscopic remission.^2^ There are many indices that are used to measure endoscopic activity, but the only validated index is the Ulcerative Colitis Endoscopic Index of Severity [UCEIS], which is independent of patient-reported symptoms^3^ and has favourable inter- and intra-rater reliability.^4–8^ Endoscopic assessment is both a treatment target and a monitoring procedure,^2^ which implies multiple endoscopies that are not only invasive and uncomfortable, but expensive to the healthcare service. As a result, the search for biomarkers that correlate with endoscopic findings is a topic of much interest to patients, clinicians and clinical trial lists.

The most useful biomarker to date, faecal calprotectin, has been shown to correlate with endoscopic and histopathologic remission with a sensitivity of 85% and specificity of 89%.^9^ It has been reported to predict relapse for those patients in remission, with both a sensitivity and a specificity of 69%.^10^ A number of other potential biomarkers have been proposed for UC including C-reactive protein^11^ levels in serum, along with myeloperoxidase^12^ and lactoferrin^13^ levels in faeces. Collecting stool samples, however, is less popular with patients than collecting blood samples.

In this prospective study, we analysed plasma samples from 40 individuals who underwent two sigmoidoscopies a median of 6 months apart. We used nuclear magnetic resonance spectroscopy [NMR] metabolomics analysis coupled with multivariate statistical techniques to determine if the metabolite profile might be used in place of endoscopy to assess disease activity. We also assessed whether the metabolite profile could be used to predict improvement or worsening in disease activity.

## 2. Methods

### 2.1. Patients

All patients had previously been diagnosed with UC according to European Crohn’s and Colitis Organisation criteria^14^ and had participated in the TrueColours UC trial.^9^ TrueColours UC involved a prospective study of 60 patients in Oxford for 6 months, during which multiple parameters were collected, including daily symptoms, fortnightly quality of life, monthly blood results and endoscopy at the start and end of the 6-month period, or in the event of a relapse. Patients who had two sigmoidoscopies at least 5 months apart with matched plasma samples at time of sigmoidoscopy and full histopathology results were included in this study. Patients with a previous colectomy were excluded. All patients underwent the same sigmoidoscopy preparation with a sodium dihydrogen phosphate dihydrate/disodium phosphate dodecahydrate enema. All patients gave written consent to participate in the study [Hampshire B Research Ethics Committee C Ref: 16/SC/0103]. All procedures performed in studies involving human patients were in accordance with the ethical standards of the institutional and/or national research committee and with the 1964 Helsinki declaration and its later amendments or comparable ethical standards. The majority [70%] of patients had no co-morbidities at the time of sampling. Of the 30% with comorbidities, these were varied: inflammatory arthritis [2], primary sclerosing cholangitis [1], osteoarthritis [3], hypertension [3], increased cholesterol [1], foraminal stenosis [1] and depression [1].

### 2.2. Clinical activity

The TrueColours programme used email prompts that contained a direct link to a questionnaire to complete the SCCAI, which assesses clinical disease activity [through questions about rectal bleeding, bowel frequency, urgency, general well-being and extraintestinal manifestations] on a daily basis. The SCCAI ranges from 0 to 19 [0 = best, 19 = worst].

### 2.3. Endoscopic activity

Each patient had two sigmoidoscopies, one at TrueColours’ entry and another towards trial completion at 4–6 months. Endoscopic results were graded by the endoscopist using the UCEIS, ranging from 0 to 8 [0 = best, 8 = worst]. For the purposes of this study, improvement in endoscopic activity was defined as a decrease in the UCEIS score of ≤1 point while worsening was defined as an increase in the UCEIS score by ≥1 point.

### 2.4. Histological activity

Biopsies were taken at each endoscopy, which were graded by a gastrointestinal histopathologist using the Nancy Index, ranging from 0 to 4 [0,1 = quiescent disease, 4 = worst].^15^

### 2.5. Medications

Medications for UC are diverse and it is common for patients to be on a combination of medications. Each patient’s medications were recorded in TrueColours UC. For the purpose of classifying these medications into groups, the following structure was used: topical rectal medications (prednisolone or 5-aminosalicylic acid [5-ASA] suppositories or enemas), oral 5-ASA, oral prednisolone, immunosupressants [azathioprine, mercaptopurine, methotrexate] and biologics [adalimumab, infliximab, vedolizumab]. A change in medication was defined as dose change, cessation or commencement of any of the above medications. No patients were taking probiotics.

### 2.6. Blood results

Blood samples were taken on the day of the sigmoidoscopy. No specific dietary instructions were given. Samples were processed as per standard laboratory procedures for haemoglobin [Hb, g/L], white cell count [WCC, ×10^9^/L], platelet count [Plt, ×10^9^/L], albumin [Alb, g/L], C-reactive protein [CRP, mg/L], ferritin [μg/L] and transferrin saturation [%]. These are henceforth referred to as blood parameters.

### 2.7. NMR sample preparation

Additional blood was collected into vacutainer lithium-heparin tubes [Becton Dickinson, product number 367375] and stored at room temperature for at least 30 min before centrifugation at 2200 × *g* for 10 min. Plasma was immediately aliquoted and stored at −80°C. For NMR analysis, the plasma samples were defrosted at room temperature and centrifuged at 100000 × *g* for 30 min at 4°C. Then, 150 μL of the plasma supernatant was diluted with 450 μL of 75 mM sodium phosphate buffer prepared in D_2_O [pH 7.4]. Samples were then centrifuged at 16000 × *g* for 3 min to remove any precipitate before transferring to a 5-mm NMR tube.

### 2.8. NMR spectroscopy

All NMR spectra were acquired using a 700-MHz Bruker AVIII spectrometer operating at 16.4 T equipped with a ^1^H [^13^C/^15^N] TCI cryoprobe. Sample temperature was regulated at 310 K. ^1^H NMR spectra were acquired using a 1D NOESY presaturation scheme for attenuation of the water resonance with a 2-s presaturation. A spin-echo Carr-Purcell-Meiboom-Gill [CPMG] sequence with a τ interval of 400 μs, 80 loops, 32 data collections, an acquisition time of 1.5 s, a relaxation delay of 2 s and a fixed receiver gain was used to supress broad signals arising from large molecular weight plasma components. CPMG spectra provide a measurement of small molecular weight metabolites and mobile side chains of lipoproteins in the plasma sample and were used for all further analysis. ^1^H correlation spectroscopy [COSY] spectra were acquired on at least one sample in each classification to aid in metabolite identification. For quality control, pooled plasma samples were spread throughout the run to monitor technical variation

### 2.9. NMR data pre-processing

Resulting free induction decays [FIDs] were zero-filled by a factor of 2 and multiplied by an exponential function corresponding to 0.30 Hz line broadening prior to Fourier transformation. All spectra were phased, baseline corrected [using a 3^rd^ degree polynomial], and chemical shifts referenced to the lactate-CH_3_ doublet resonance at δ = 1.33 ppm in Topspin 2.1 [Bruker]. Spectra were visually examined for errors in baseline correction, referencing, spectral distortion or contamination and then exported to ACD/Labs Spectrus Processor Academic Edition 12.01 [Advanced Chemistry Development, Inc.]. The regions of the spectra between 0.08–4.20 and 5.20–8.50 ppm were divided in to 0.02-ppm width ‘buckets’ and the absolute value of the integral of each spectral bucket was Pareto scaled [termed ‘bucket integrals’]. Resonances were assigned by reference to literature values^16,17^ and the Human Metabolome Database^18^ and further confirmed by inspection of the 2D spectra, spiking of known compounds and 1D-TOCSY spectra.

### 2.10. Statistical analysis

The bucket integrals were imported into R software [R Foundation for Statistical Computing].^19^ Due to the size of the datasets, univariate analysis with multiple comparison correction is not appropriate and multivariate strategies were employed. All multivariate analysis was carried out using in-house R scripts and the *ropls* package.^17^

Principal component analysis [PCA] was employed to investigate correlated variation in the NMR spectra in an unsupervised [blinded] manner. Orthogonal partial least squares discrimination analysis [OPLS-DA] was employed to investigate these separations in greater detail, produce predictive mathematical models and identify the metabolites driving the discrimination between classes.

OPLS-DA models were optimized by internal 7-fold cross-validation. The quality of classification was assessed using a 10-fold external cross-validation scheme with 1000 repetitions, correcting for unequal class sizes. This validation scheme involves multiple iterations of splitting the data into training and testing sets, which ensures that any discrimination observed in the models cannot have occurred by chance. The training data are used to estimate the model parameters and learn the underlying discriminatory patterns between the classes under consideration, whereas the independent test set is employed to assess the accuracy and generalizability of the trained models in the ensemble. We quantified the outcome of the cross-validation by calculating the accuracy, sensitivity and specificity of each model from the predicted classifications of each external independent test set. It is important to appreciate that the classifier [OPLS-DA] was blinded to each test set when training each model. This validation scheme tends to avoid over-fitting and helps to assess the generalizability of the model to previously unseen datasets. For an exhaustive discussion on validation of this approach see Arlot and Celisse.^20^ These values were compared with those of a null distribution [obtained from randomly permuting the classes] using the two-sided Kolmogorov–Smirnov test [significant at *p* ≤ 0.05].

## 3. Results

### 3.1. Multivariate analysis discriminates between patients with UCEIS scores ≤3 and >3 using the plasma metabolite profile alone

Of the 40 patients in the study, 23 [58%] were female, median age was 40 years [range 17–62 years], median disease duration was 6 years [range 1.2 months to 46 years], and extent of disease was 8/40 proctitis, 15/40 left-sided colitis, 15/40 extensive disease and 2/40 unsure. The median UCEIS score in the worst affected area at baseline was 3 [range 0–7].

**Figure 1.**
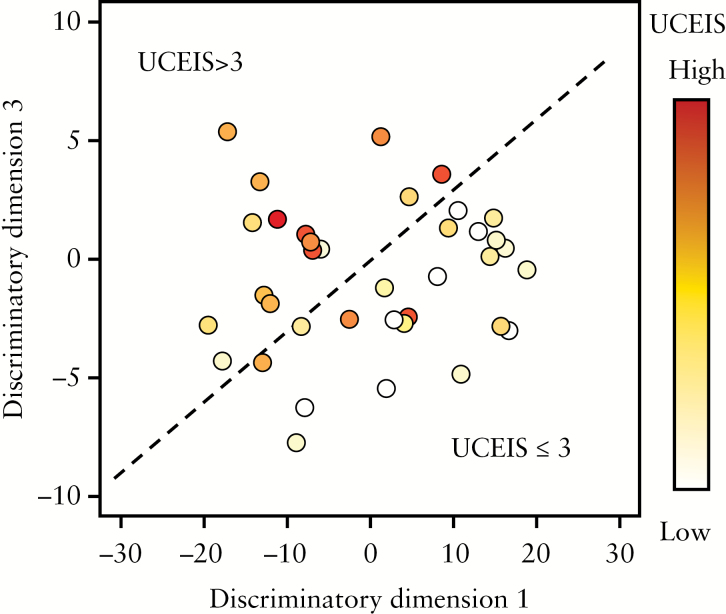
PCA scores plot illustrating spontaneous separation of plasma NMR metabolite profiles according to high [>3] or low [≤3] UCEIS, forming two distinct clusters.

PCA was used to identify which individuals in the cohort had similar baseline plasma NMR metabolite profiles in an unsupervised [blinded] manner. Investigation of the resulting PCA scores plot [Figure 1] revealed that the plasma metabolite profiles [each point in the plot represents an individual patient’s NMR spectrum] spontaneously segregated UCEIS scores ≤3 from scores >3. A comparison of the demographics of patients with low [≤3] and high UCEIS [>3] is shown in Table 1. This separation indicates that there are significant differences in the plasma metabolites of patients with low UCEIS compared to high UCEIS. This was confirmed using OPLS-DA.

**Table 1.** Comparison of the demographics of patients with low and high UCEIS.

	Low UCEIS	High UCEIS
Number of patients	24	16
Gender [% female]	58	56
Age, median [range], years	42 [17–62]	37 [22–56]
Body mass index, median [range]	25.0 [18.7–37.1]	23.1 [18.7–31]
Disease duration, median [range], years	7 [0.1–46]	4 [0.1–18]
Time between sigmoidoscopy, median [range], months	6 [5–7]	5 [5–7]
Proctitis/left-sided colitis/extensive colitis, %	17/29/50	25/50/19
Co-morbidities: none/present [%] Total patient numbers with co-morbidity [*n*]	63/37 2 Osteoarthritis 2 Inflammatory arthritis 2 Hypertension 1 Increased cholesterol 1 Foraminal stenosis 1 Depression	81/19 1 Osteoarthritis 1 Hypertension 1 Primary sclerosing cholangitis
Never smoked,/ex-smoker/current smoker, %	46/50/4	69/31/0
Nil medications, %	4	6
Topical 5-ASA, %	21*	56
Topical steroid, %	4	13
Oral 5-ASA, %	54*	81
Oral steroid, %	17	19
Immune suppressant, %	29	19
Anti-TNF, %	13	6
Vedolizumab, %	17	25
Cyclosporin, %	4	0
Probiotics, %	0	0

All 40 patients in the study cohort are included. *Significant difference in distribution [chi-squared, *p* < 0.05]. 5-ASA, 5-aminosalicylic acid; TNF, tumour necrosis factor.

**Figure 2.**
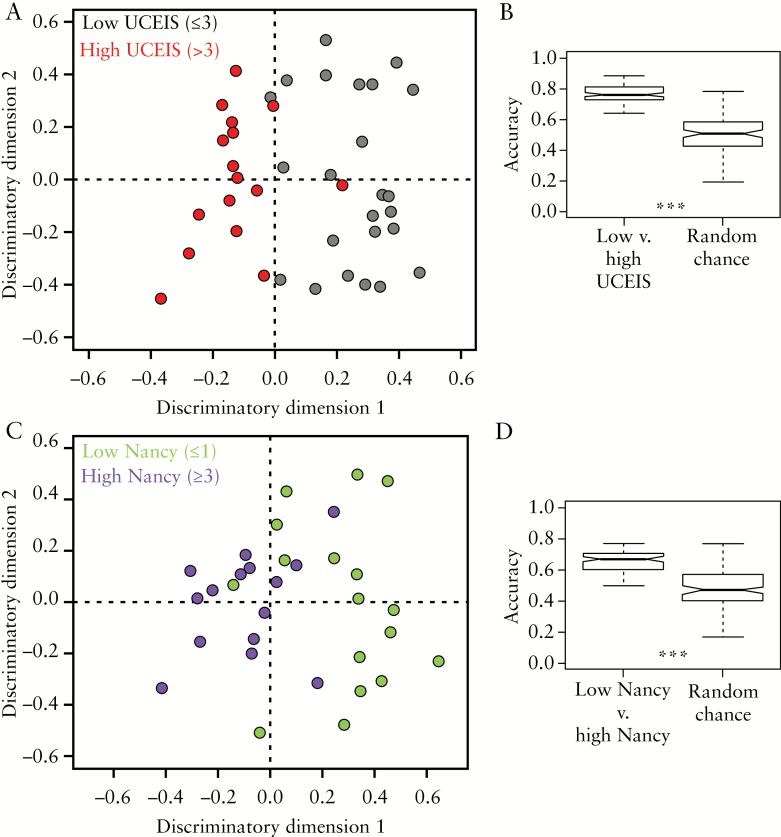
[A] OPLS-DA scores plot discriminating between high [>3, red] and low [≤3, black] UCEIS. [C] OPLS-DA scores plot discriminating between high [≥3, purple] and low [≤1, green] Nancy. [B, D] The accuracy of the ensemble of OPLS-DA models is significantly higher than the null distribution. Kolmogorov–Smirnov test, ***p < 0.001.

The OPLS-DA model was able to discriminate between plasma samples from patients with low and high UCEIS with an accuracy, sensitivity and specificity of 77 ± 5, 82 ± 7 and 82 ± 7%, respectively, using the plasma NMR metabolite profile alone [Figure 2A]. To ensure that this separation did not occur by chance, a 10-fold cross-validation scheme with repetition was employed as described in detail in the Methods. A permutation test was then used to confirm that the performance of the OPLS-DA model was significantly better than the null distribution [produced by randomly assigning the classifications]. Figure 2B illustrates that the accuracy of the low UCEIS vs high UCEIS ensemble of OPLS-DA models is significantly greater than that achieved by random chance, rigorously validating the separation observed.

**Figure 3.**
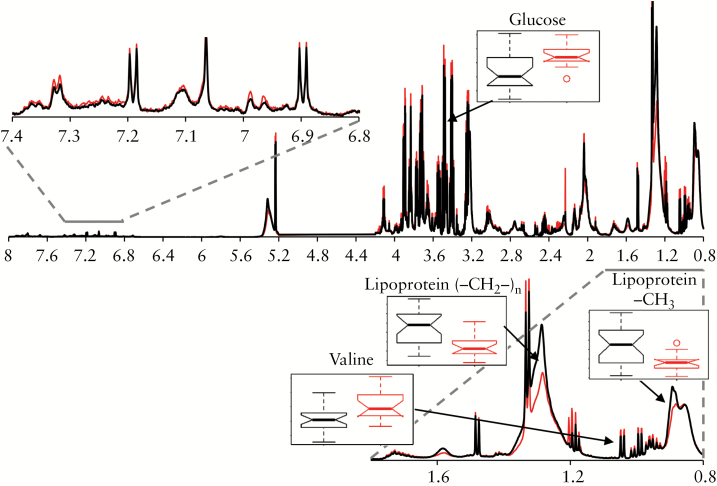
Average ^1^H CPMG NMR resonance intensity of all plasma samples from patients with high [>3, red] and low [≤3, black] UCEIS. Box plots illustrate significant differences in the NMR spectral integrals of a selection of metabolites selected as significant discriminators by the OPLS-DA models.

The NMR variables driving the discrimination in the OPLS-DA models included several resonances from mobile lipoprotein side-chains [-CH_3_, (-CH_2_-)_n_, βCH_2_, unsaturated lipid, and =CH-CH_2_-CH_2_-], all of which were decreased in high UCEIS plasma samples compared to low [Table 2]. In contrast, isoleucine, valine, glucose and *myo*-inositol were significantly increased in plasma from individuals with high UCEIS compared to those with low UCEIS [Figure 3]. NMR spectral integrals of all significant metabolites selected by the OPLS-DA models are shown in Supplementary Figure S2.

**Table 2.** Discriminatory variables driving the separation observed in the OPLS-DA models.

	High UCEIS vs low UCEIS	Worsening UCEIS vs improving UCEIS	High Nancy vs low Nancy
**Lipoproteins**			
-CH_3_	↓ [–25%]	↑ [31%]	↓ [–13%]
(-CH_2_-)_n_	↓ [–36%]	↑ [49%]	↓ [–20%]
βCH_2_	↓ [–37%]	↑ [57%]	↓ [–22%]
Unsaturated lipid	↓ [–26%]	↑ [22%]	↓ [–17%]
=CH-CH_2_-CH_2_-	↓ [–15%]	–	↓ [–9%]
**Branched chain amino acids**			
Isoleucine	↑ [18%]	–	–
Valine	↑ [21%]	–	↑ [5%]
**Other**			
Glucose	↑ [15%]	–	↑ [9%]
*myo*-Inositol	↑ [26%]	↑ [25%]	↑ [26%]
**Other amino acids**			
Lysine	–	↓ [–10%]	–
Histidine	–	↓ [–24%]	–
Phenylalanine	–	↓ [–17%]	–
Tyrosine	–	↓ [–23%]	–

Increases and decreases in metabolite concentrations in the high [>3] UCEIS cohort when compared to the low [≤3] UCEIS cohort, in the worsening compared to the improving UCEIS cohorts, and the high [≥3] compared to the low [≤1] Nancy cohorts are indicated by arrows. Metabolites not selected by the OPLS-DA model are indicated by a dash.

The same metabolite profile, with the omission of isoleucine [Table 2], was able to distinguish between plasma samples from patients in histological remission [Nancy ≤ 1] and those with moderate to severe inflammation [Nancy ≥ 3] with an accuracy, sensitivity and specificity of 65 ± 6, 73 ± 5 and 74 ± 5%, respectively. To separate those samples with truly active disease [Nancy 3 and 4] from those without active disease [Nancy 0 and 1], eight patients with mildly active histological activity [Nancy 2] were removed from the analysis in this instance. Although the metabolite profile is less accurate at discriminating between Nancy score than UCEIS, the OPLS-DA models are significantly better than chance [Figure 2C, D].

### 3.2. NMR plasma metabolite profile predicts improvement or worsening in UCEIS up to 7 months from sample collection

**Figure 4.**
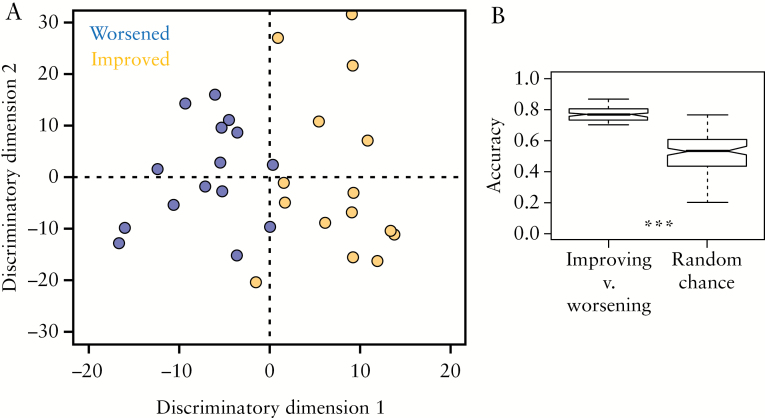
[A] OPLS-DA scores plot discriminating between baseline plasma samples from patients whose UCEIS increased [worsened, blue] or decreased [improved, yellow] over the course of the study. [B] The accuracy of the ensemble of OPLS-DA models is significantly higher than the null distribution, indicating that the discrimination did not occur by chance. Kolmogorov–Smirnov test, ***p < 0.001.

Each patient included in this study had two sigmoidoscopies, a median 6 months apart [range 5–7 months]. Over this time period, 19 patients improved [a decrease in UCEIS by at least one point], six had no change in UCEIS and 15 worsened [an increase in UCEIS by at least one point]. To determine if the plasma metabolite profile was predictive of disease course, a new ensemble of OPLS-DA models was produced using only the baseline plasma samples from those individuals who improved or worsened over the duration of the study. The demographics of patients in the improved as compared to the worsened group are given in Table 3. The OPLS-DA model [Figure 4A], using only the metabolite profile of the baseline [month 0] plasma sample, was able to identify which patients improved or worsened in the 5–7 month follow up with an accuracy, specificity and sensitivity of 74 ± 4, 81 ± 7 and 81 ± 8%, respectively. The accuracy of the ensemble of models was significantly greater than random chance [Figure 4B] validating the separation observed. Of the seven patients with no change in UCEIS over the course of the study, the discriminatory model predicted two to be worsening and correctly identified the remaining five as borderline between the two classes, resulting in an accuracy of 71% in this group [Supplementary Figure S1].

**Table 3.** Comparison of the demographics of patients improved and worsened over the course of the study.

	Improved	Worsened
Number of patients	18	15
Gender [% female]	42	44
Age, median [range], years	39 [21–56]	38 [19–62]
Body mass index, median [range]	23.1 [18.7–37.1]	24.9 [18.7–35.0]
Disease duration, median [range], years	7 [0.1–26]	6 [0.1–46]
Time between sigmoidoscopy, median [range], months	6 [5–7]	6 [5–7]
Proctitis/left-sided colitis/extensive colitis, %	28/39/33	20/20/60
Co-morbidities: none/present, % Total patient numbers with co-morbidity [*n*]	67/33 1 Osteoarthritis 1 Inflammatory arthritis 3 Hypertension 1 Increased cholesterol	67/33 2 Osteoarthritis 1 Inflammatory arthritis 1 Depression 1 Primary sclerosing cholangitis
Never smoked,/ex-smoker/current smoker, %	67/33/0	40/53/7
Nil medications, %	6	7
Topical 5-ASA, %	50	20
Topical steroid, %	6	7
Oral 5-ASA, %	72*	53
Oral steroid, %	22	20
Immune suppressant, %	13	20
Anti-TNF, %	11	13
Vedolizumab, %	11	20
Cyclosporin, %	6	0
Probiotics, %	0	0

Seven patients with stable disease, defined by no change in UCEIS, were excluded. *Significant difference in distribution [chi-squared, *p* < 0.05]. 5-ASA, 5-aminosalicylic acid; TNF, tumour necrosis factor.

**Figure 5.**
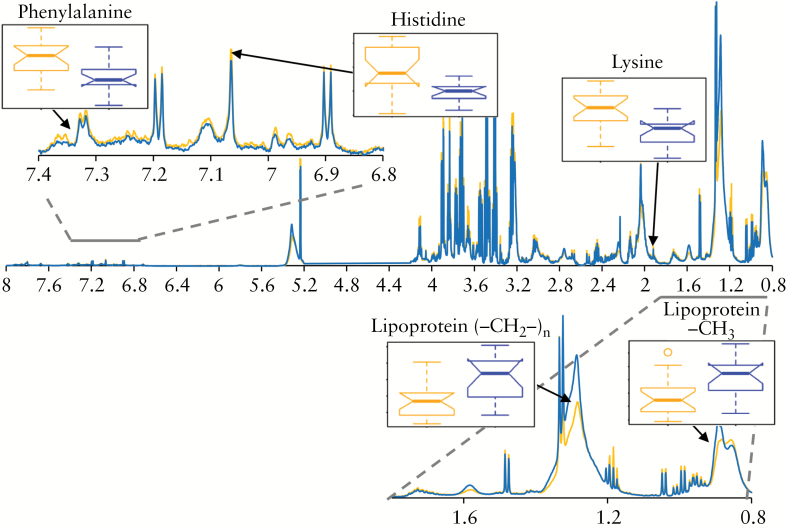
Average ^1^H CPMG NMR resonance intensity of all baseline plasma samples from patients whose UCEIS increased [worsened, blue] or decreased [improved, orange] over the course of the study. Box plots illustrate significant differences in the NMR spectral integrals of a selection of metabolites selected as significant discriminators by the OPLS-DA models.

In contrast to the low vs high UCEIS model, neither isoleucine, valine nor glucose were selected as discriminatory variables for the disease outcome model above, confirming that the metabolite profile which distinguishes low from high UCEIS is distinct from the metabolite profile which predicts improved/worsened disease. Lipoprotein resonances [-CH_3_, (-CH_2_-)_n_, βCH_2_ and unsaturated lipid] and *myo*-inositol were increased in the worsened cohort when compared to the improved cohort [Figure 5]. In addition, lysine, histidine, phenylalanine and tyrosine were significantly decreased in the worsened group compared to the improved group [Table 2]. NMR spectral integrals of all significant metabolites selected by the OPLS-DA models are shown in Figure S3.

### 3.3. NMR plasma metabolic profile is independent of standard blood measurements and SCCAI

None of the blood parameters measured [Hb, WCC, Plt, CRP, Alb, ferritin or transferrin] were able to distinguish between low and high UCEIS, or to predict which patients improved or worsened. In addition, no significant linear correlations were observed between any of the clinical chemistry parameters measured and the UCEIS, SCCAI or any of the NMR metabolite levels. Finally, addition of the clinical chemistry data to the multivariate analysis did not improve accuracy. Taken together, these results suggest that the discriminatory metabolite profiles identified [Table 2] are independent of plasma Hb, WCC, Plt, CRP, Alb, ferritin and transferrin levels.

**Figure 6.**
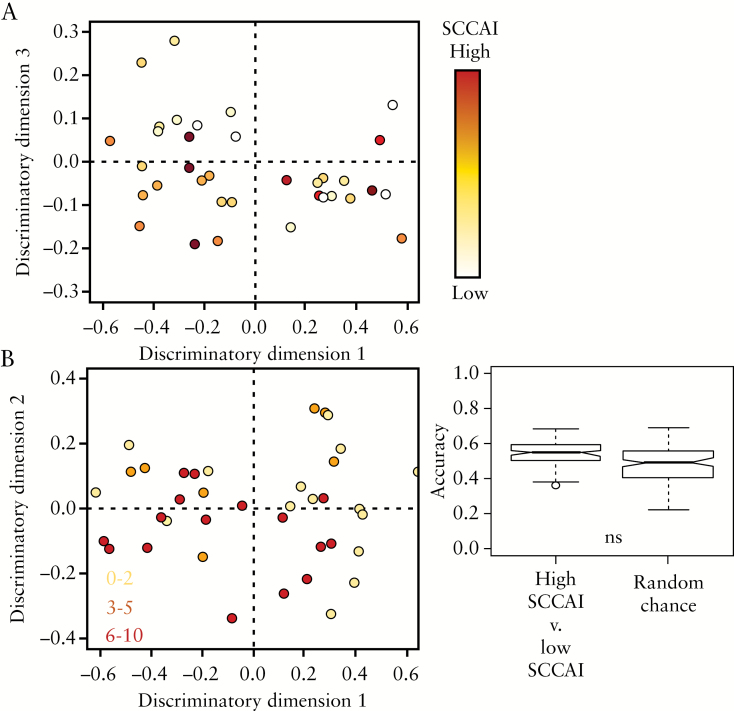
[A] PCA scores plot representing the distribution of SCAII scores within the cohort. In contrast to the UCEIS, no spontaneous clustering as a result of SCAII is observed. [B] OPLS-DA scores plot is unable to discriminate between the plasma NMR metabolite profiles based on SCAII. The accuracy of the OPLS-DA models is not significantly better than random chance.

Unlike the UCEIS-PCA scores plot [Figure 1], the NMR metabolite profiles do not spontaneously cluster according to SCCAI. The PCA scores plot [Figure 6A] shows that the high SCCAI metabolite profiles are equally distributed among the low SCCAI metabolite profiles. As a result, OPLS-DA is unable to produce a valid model according to SCCAI classification [Figure 6B] showing that the metabolite profile measured by NMR is independent of SCCAI.

### 3.4. No potential confounding factors investigated significantly impacted the plasma metabolic profiles

To confirm that the separations observed in the OPLS-DA models were not due to any known potential confounding factors, patient demographics were compared between classes in each OPLS-DA model. The demographics of the low UCEIS group were compared to the high UCEIS group [Table 1] using a chi-squared test for independence [significant at *p* < 0.05]. Likewise, the demographics of the patients who improved were compared to those who worsened [Table 3]. No significant differences were observed in the age, gender, length of follow up, disease duration or extent of disease [proctitis/left-sided colitis/extensive colitis] between the groups studied here. A detailed investigation of all potential confounders listed in Tables 1 and 3 was undertaken; multivariate regression analysis [OPLS-DA] was not significant and no correlations were observed with the metabolites measured for any of the potential confounders. Furthermore, addition of confounders as variables in the OPLS-DA analysis did not significantly improve accuracy, confirming that the separations observed are not driven by potential confounding factors.

**Figure 7.**
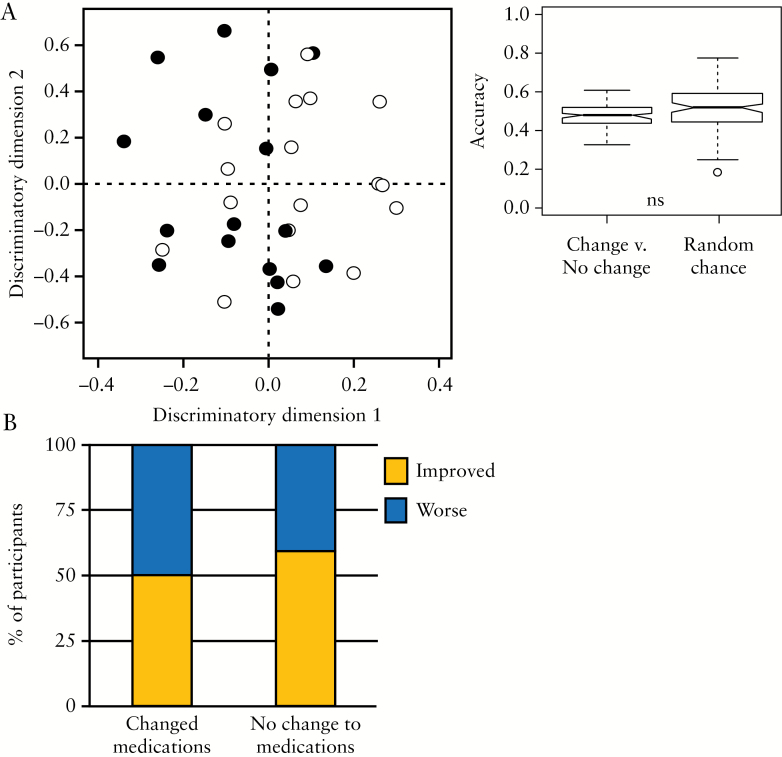
[A] OPLS-DA scores plot is unable to discriminate between the metabolite profiles of patients who changed medication [white] during the course of the study compared to those with no change in medication [black]. [B] Box plot of the percentage of patients who improved/worsened following a change in medication compared to those who stayed on the same medications over the course of the study.

Although all potential confounders in Tables 1 and 3 were investigated in detail, particular attention was paid to any effect of medications on the metabolite profile, because 94% patients in the study were on therapy with 60% were prescribed more than one medication at baseline. However, only 13% patients were prescribed the same combination of medications at any given time, so the separation between classes observed above is not a result of different treatment combinations. Over the course of the study, a change in medication was made to 48% of patients, but OPLS-DA was unable to separate those who changed medication from those who did not [Figure 7A]. There was no appreciable correlation between those who changed medications and those who improved [Figure 7B].

**Figure 8.**
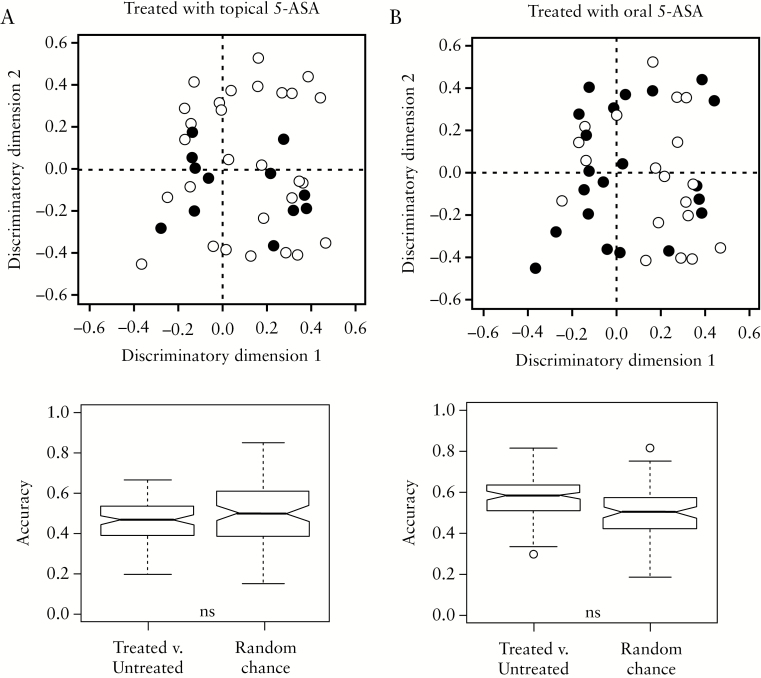
The plasma NMR metabolite profiles of patients treated with [A] oral 5-ASA and [B] oral 5-ASA [black] are evenly spread through the untreated samples [open circle] in the OPLS-DA scores plots.

To confirm that the differences observed in the plasma metabolite profiles were not due to individual medications, the number of patients in each class [improved, worsened, low UCEIS and high UCEIS] taking each therapy was investigated [Tables 1 and 3]. No significant difference between classes was observed with the exception of topical 5-ASA, which was prescribed to more patients in the high UCEIS class than the low UCEIS class, and oral 5-ASA, which was prescribed to more in the improved and high UCEIS classes compared to the worsened and low UCEIS classes, respectively. Despite this, OPLS-DA was unable to build a model to separate untreated vs treated for either topical [Figure 8A] or oral [Figure 8B] 5-ASA. This was the case for all medications listed in Table 1 [data not shown]. In addition, no correlation was observed between any of the metabolite variables and medications. Inclusion of medications as variables in the OPLS-DA models did not significantly improve accuracy, and no known drug metabolite peaks were observed in the NMR spectra. As a result, we conclude that the medications listed in Table 1 had no appreciable impact on the NMR metabolite profiles or multivariate analysis employed.

## 4. Discussion

For optimal management of UC, a non-invasive test that can be performed frequently and provide a measure of endoscopic disease severity is needed. Faecal calprotectin is widely used, but the test is relatively expensive, so research into biomarkers continues.

NMR metabolomics of plasma is a relatively non-invasive and cost-effective technique capable of quantifying a broad spectrum of metabolites simultaneously. Each experiment takes only a few minutes and produces a metabolite profile of a patient at a given point in time. As any perturbation to homeostasis results in alteration of the metabolome, the metabolic profile can be used to rapidly measure the underlying disease state. Many reports have illustrated differences between the metabolic profiles of patients with inflammatory bowel disease [IBD] and controls in faecal extracts,^21^ intestinal tissue biopsies,^22,23^ urine^24–27^ and blood.^25,27,28^ However, only a small number have investigated UC in isolation^29,30^ or compared the metabolite signatures of active and inactive disease,^31–33^ and the majority of studies analyse samples retrospectively. The use of different disease severity indices and the mixing of patients with Crohn’s disease with those who have UC creates further challenges to interpreting metabolomics data in IBD.

This study presents data from a cohort of 40 UC patients who were followed up prospectively over a median 6 months. The data demonstrate that the plasma NMR metabolite profile is able to distinguish between patients with low [UCEIS ≤ 3] and high [UCEIS > 3] endoscopic activity with an accuracy of 77 ± 5%, confirming the potential of NMR analysis of blood in monitoring UC. In this cohort, we were unable to distinguish between each UCEIS level, suggesting that the metabolite profile does not, as yet, provide the detailed assessment achieved by endoscopy. While future work investigating a larger cohort may allow more refined stratification, the ability to stratify patients into two groups correlating with high and low activity would be of significant clinical benefit, as plasma NMR can be performed when other blood samples are being taken at outpatient follow up. In addition, future work will determine if endoscopic remission [UCEIS 0, which best correlates with histological remission] can be discriminated from activity [UCEIS > 0].

The variables driving this discrimination between low and high UCEIS included several lipoprotein resonances [-CH_3_, (-CH_2_-)_n_, βCH_2_, =CH-CH_2_,-CH_2_- and unsaturated lipid], all of which were decreased in plasma samples from patients with high endoscopic activity compared to those with low activity. This may be a result of alterations in lipoprotein metabolism as a result of increased inflammatory activity in patients with high UCEIS. Previous reports have demonstrated altered lipid profiles in patients with IBD compared to controls; decreased total cholesterol [Tot-c], low-density lipoprotein cholesterol [LDL-c], and high-density lipoprotein cholesterol [HDL-c] along with increased total triglycerides [TG] have been observed.^34–36^ In addition, low Tot-c and high TG have been found to associate independently with the number of hospitalizations and IBD operations, respectively.^37^ However, it should be noted that the extent of lipid profile alterations in IBD and UC in particular is not fully understood. There are conflicting reports, with other studies illustrating increases or no alteration in plasma lipoprotein levels.^38,39^

Increases in the concentration of isoleucine, valine, glucose and *myo*-inositol were observed in plasma samples from patients with high endoscopic activity [UCEIS ≥ 3]. High glucose levels have been previously noted in the faeces,^40^ colonic mucosa^41^ and serum^25,29^ of patients with IBD relative to controls. While prednisolone can result in increased glucose levels, it should be noted that there was no significant difference in the number of our patients on steroids in the high UCEIS group compared to the low UCEIS group [Table 1]. A link between amino acid composition and UC pathogenesis has been speculated and amino acid supplementation has been explored as therapy for UC; previous studies have described changes in the amino acid profile in UC, with isoleucine serum levels increased relative to controls.^42^

The same pattern of metabolite concentration changes responsible for the discrimination between low and high endoscopic activity also drove the discrimination between histological remission and moderate/high histological inflammation [Table 2]. However, while the OPLS-DA models were significantly better than random chance, their accuracy in identifying patients with low or high Nancy scores was lower than for the UCEIS. That the plasma metabolite profile is more strongly related to endoscopic activity than to histological activity in this study may be a consequence of sample size or indicate that the metabolite profile reflects the global disease burden.

To the best of our knowledge, only two publications have investigated the prognostic potential of metabolic profiling. The ^1^H NMR spectra of mucosal biopsies have been used to distinguish 12 patients with inactive UC [defined as an endoscopic Mayo score of 0–1] from a cohort of 33 who were retrospectively characterized as having a flare-up within 6 months of sample collection.^30^ In the other study, also retrospective, low plasma histidine levels were found to associate with relapse within 1 year in patients with UC in remission at the time of sampling [where remission was defined loosely as a Lichtiger clinical activity index <5 and activity ≥5].^43^ Recently, serum neutrophil-related markers were shown to associate with mucosal healing following adalimumab treatment.^44^

The data reported here, from a prospective study, demonstrate that plasma samples from patients who improved and worsened over a median 6-month period can be discriminated using their baseline plasma metabolite profile alone with an accuracy of 74 ± 4%. Here, improvement was defined in endoscopic [a UCEIS change ≥1] and not clinical terms. The size of this cohort precluded the investigation of larger changes in the UCEIS. The prognostic OPLS-DA models revealed that patients who worsened over the study period had higher plasma lipoprotein [-CH_3_, (-CH_2_-)_n_, βCH_2_ and unsaturated lipid] and *myo*-inositol levels at baseline when compared with patients whose endoscopic activity improved, along with decreased lysine, histidine, phenylalanine and tyrosine concentrations. It is possible that the decreased amino acid levels identified here prior to increased disease activity could be a result of perturbations of amino acid metabolic pathways or simply the result of malabsorption due to low-level increased inflammation that was undetectable by endoscopy at baseline. The decreased histidine levels in the worsening group reported here are consistent with the observation that low plasma histidine levels are associated with relapse.^43^ It should also be noted that no correlation was observed between medication and those who improved over the course of the study [Figure 7]. Interestingly, the metabolite pattern for prognosis [discriminating between those who improved vs worsened] was distinct from the pattern which distinguished between low and high endoscopic activity at the time of sampling. While changes in plasma lipoprotein levels were identified in both models, the specific resonances selected and fold-change patterns differ. A significant decrease in the =CH-CH_2_-CH_2_- resonance was observed in baseline plasma from patients with high endoscopic activity compared to low, while no significant difference in this lipoprotein resonance was observed when comparing those who worsened to those who improved. Furthermore, the endoscopic activity model [comparing low vs high UCEIS] revealed significant differences in isoleucine, valine and glucose plasma concentrations, but there were no significant differences in these metabolites in the prognostic model [comparing improved vs worsened]. Conversely, significant decreases in lysine, histidine, phenylalanine and tyrosine were observed in the prognostic model which were not observed in the model of endoscopic activity. This suggests that the metabolite pattern for determining UC severity [i.e. endoscopic activity] at a point in time is distinct from the pattern predicting disease activity over a period of time [6 months]. This is consistent with the fact that UCEIS does not provide prognostic information and that the improved/worsened groups do not contain the same individuals as the low/high UCEIS (i.e. the improved and worsened) groups each contain patients with low and high UCEIS at baseline.

The metabolite profile was unable to discriminate UC plasma samples based on clinical symptoms alone, using the SCCAI. The SCCAI is influenced by patient well-being and mood, so other factors [unrelated to UC] can result in an elevated SCCAI. This suggests that the metabolite profile identified here provides a signature of the underlying UC pathophysiology. A panel of blood parameters was measured on each blood sample, none of which correlated with disease severity [defined by either UCEIS or SCCAI] and were not predictive of improvement/worsening over time. As a result, the metabolic profiles identified here are independent of the blood parameters measured. Furthermore, the metabolite profile was confirmed to be independent of all potential confounding factors listed in Tables 1 and 3, and the separations observed in the OPLS-DA models could not be attributed to different medications or changes to medications over the course of the study.

Although no biofluid-based test is available to measure UC severity or predict disease course, several biomarkers have been identified which are used as an adjunct to clinical evaluation. However, no single biomarker is sensitive and specific enough to act in isolation and so a combination of markers and interpretation in the context of other clinical features by an experienced clinician is required. Furthermore, there is currently no method to predict both improvement and worsening over the extended time period demonstrated here. The metabolomics approach is advantageous as it is cost effective, allows a range of metabolites to be measured simultaneously with a single test, and the combined pattern of variables can have greater accuracy than a single biomarker in isolation. Calprotectin has been shown to correlate with endoscopic remission with an accuracy of up to 86%,^9^ which is in contrast with the NMR metabolite profile which is able to discriminate between low and high UCEIS scores with an accuracy of 77 ± 5%. Ongoing work to validate these results in a larger cohort will investigate the accuracy of the metabolite profile in identifying endoscopic remission. However, the NMR metabolite profile may outperform faecal calprotectin when predicting disease course. Calprotectin has been shown to predict relapse [for those patients in remission] with an accuracy of 69%, while the NMR metabolite profile is able to predict patients who improved or worsened over 7 months with an improved accuracy of 74 ± 4%. Calprotectin may also provide a surrogate marker for mucosal healing, but, unlike the metabolomic approach, the variation in the absolute inter- and intra-individual calprotectin levels is likely to be a confound when probing a graded response. Very low faecal calprotectin might be used to aid decision-making about stopping therapy, although there are no data on the relevant calprotectin thresholds. In a small controlled study, the number of individuals with faecal calprotectin levels below 50 μg/g stool were greater in those randomized to an increased dose of mesalazine.^45^ However, the lack of specificity of calprotectin for IBD and the requirement for thresholding make this approach unsuitable as a surrogate marker of mucosal healing on an individual basis for routine use. The data presented here provide proof of principle that NMR analysis of plasma has the potential to act as a surrogate for endoscopic activity at a given point in time and thereby provides a means to monitor patients more frequently than is practicable by sigmoidoscopy. On the basis of this result, the use of larger cohorts is now desirable and may lead to further refinement of the metabolite profiles leading to an increase in both diagnostic and predictive accuracy.

## Funding

This work was supported by the Multiple Sclerosis Society [grant number 59 to FP]; MRC Confidence in Concept Fund MC_PC_15029; the Ministry of Health, Singapore through the National Medical Research Council Research Training Fellowship [NMRC/Fellowship/0038/2016 to TY]; and TrueColours UC was supported by the Norman Collisson Foundation, Abbvie Pharmaceuticals, Buhlmann Laboratories and the British Research Council. AS was supported by an NIHR Research Professorship and Wellcome Investigator Award.

## Conflict of Interest

ST: led the development of the UCEIS and was a co-author on the paper describing the Nancy Index, but had no other conflicts in this study. All other authors declare no conflicts of interest.

## Supplementary Material

Supplementary Information
