# Supplementary material for: Plasma Nuclear Magnetic Resonance Metabolomics Discriminates Between High and Low Endoscopic Activity and Predicts Progression in a Prospective Cohort of Patients With Ulcerative Colitis
Source: J Crohns Colitis. 2018 Jul 17;12(11):1326–37. doi: 10.1093/ecco-jcc/jjy101 (PMC6403054; doi:10.1093/ecco-jcc/jjy101)
Supplement: Supplementary Information [file jjy101_suppl_supplementary_information.docx]

**Supplementary Information: Plasma Nuclear Magnetic Resonance metabolomics discriminates between high and low endoscopic activity and predicts progression in a prospective cohort of patients with ulcerative colitis**

Fay Probert^1^, Alissa Walsh^2^, Marta Jagielowicz^3^, Tianrong Yeo^1,4^, Timothy D. W. Claridge^5^ , Alison Simmons^3^, Simon Travis^2^, Daniel C Anthony^1^.

1. Department of Pharmacology, University of Oxford, Mansfield Road, Oxford, UK
2. Translational Gastroenterology Unit, Oxford University Hospitals NHS Foundation Trust, Oxford, UK
3. Weatherall Institute of Molecular Medicine, Oxford, UK
4. Department of Neurology, National Neuroscience Institute, 11 Jalan Tan Tock Seng, Singapore
5. Department of Chemistry, University of Oxford, Chemistry Research Laboratory, Mansfield Road, Oxford, UK.

**Short title:** NMR metabolomics predicts endoscopic activity in a prospective UC study.

**Correspondence to:**

Clinical: Simon Travis, Translational Gastroenterology Unit, Oxford University Hospitals NHS Foundation Trust. Email: [simon.travis@ndm.ox.ac.uk](mailto:simon.travis@ndm.ox.ac.uk)

Analysis and interpretation: Daniel Anthony, Department of Pharmacology, University of Oxford. Email: daniel.anthony@pharm.ox.ac.uk


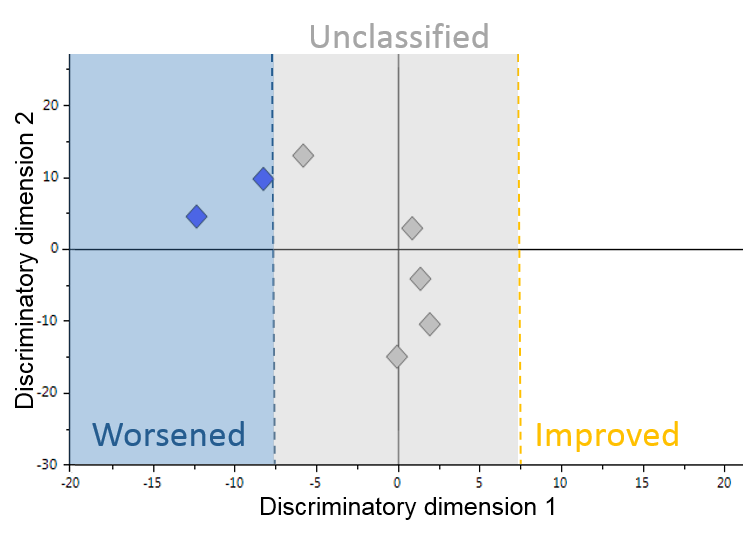


Figure SI1. Predicted classification of metabolite profiles from patients with no change in UCEIS using the discriminatory OPLS-DA model trained on worsened and improved metabolite profiles. The OPLS-DA model correctly identifies 5 of the metabolites profiles as un-classified; they neither improved nor worsened over the course of the study. Two metabolite profiles were incorrectly predicted as worsening resulting in an accuracy of 71% in this subset.


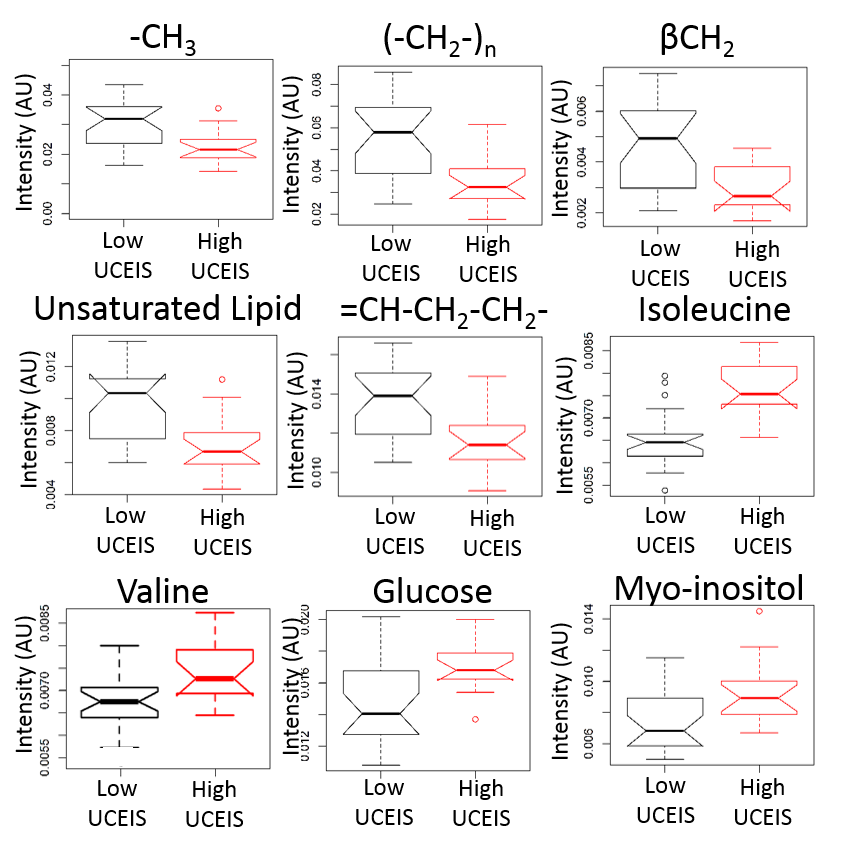


Figure SI2. Spectral intensity of all significant metabolites (determined by OPLS-DA) separating high from low UCEIS.


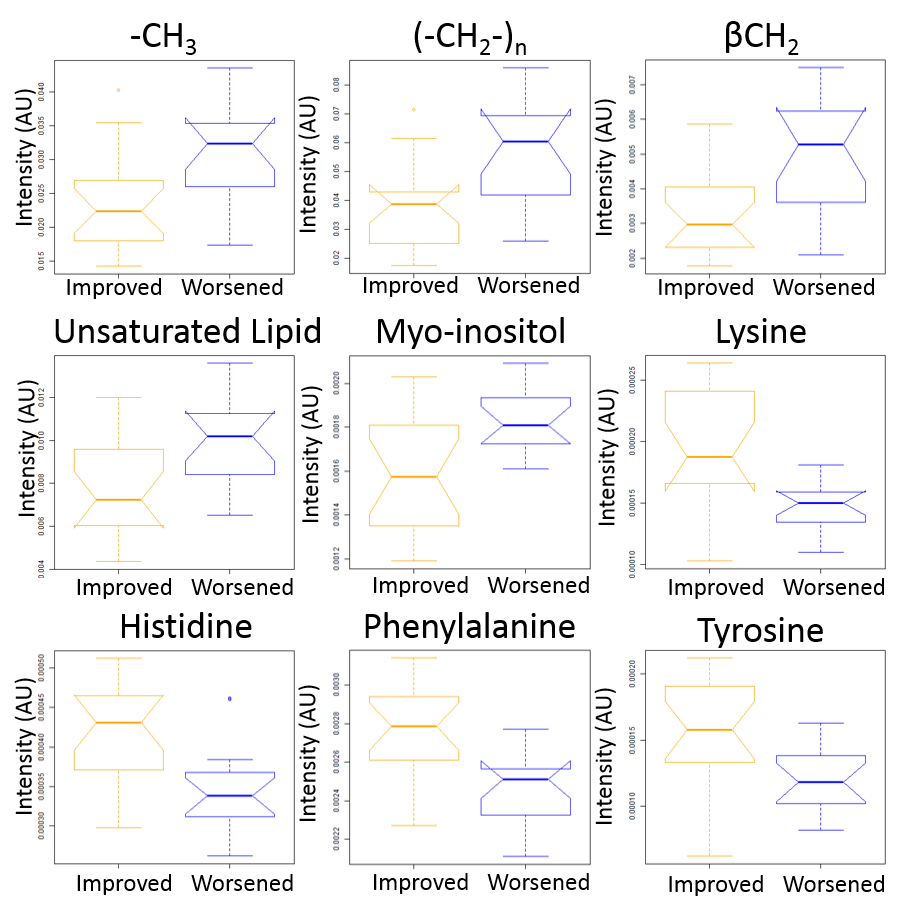


Figure SI3. Spectral intensity of all significant metabolites (determined by OPLS-DA) separating improved UCEIS from worsened UCEIS.
